# Evaluation of Different Attractants for Monitoring and Mass Trapping of *Rhagoletis batava* (Hering) in Organic Sea Buckthorn Plantations

**DOI:** 10.3390/insects16121248

**Published:** 2025-12-10

**Authors:** Małgorzata Tartanus, Witold Danelski, Ewa Maria Furmańczyk, Aya El Meziane, Eligio Malusà

**Affiliations:** 1 The National Institute of Horticultural Research, 96-100 Skierniewice, Poland; witold.danelski@inhort.pl (W.D.); ewa.furmanczyk@inhort.pl (E.M.F.); aya.elmeziane@inhort.pl (A.E.M.); 2 Council for Agricultural Research and Economics (CREA), Center for Viticulture and Enology, 14100 Asti, Italy

**Keywords:** attractants, ammonium phosphate solution, homemade trap, mass trapping, *Rhagoletis batava*

## Abstract

Farmers’ interest in producing sea buckthorn (*Hippophae rhamnoides* L.), a berry species with a high nutraceutical value, has increased in Poland and several other European countries, particularly under organic management. However, the expansion of the crop area has been paralleled by a higher occurrence particularly of the sea buckthorn fruit fly (*Rhagoletis batava* Hering, 1958), a tephritid species potentially causing severe damage to the berries. Trials to evaluate the mass trapping efficacy of various kinds of traps were carried out for up to five years in different locations, testing several attractants known to be effective with other fruit fly species. A commercial cone trap with an attractant designed for *Ceratitis capitata* and a homemade plastic bottle trap containing a 4% ammonium phosphate solution were found to be the most effective in attracting the flies in a preliminary trial and were further tested in various orchards for four years. Improved attractants were also assessed, together with the ammonium phosphate solution, but resulted in a limited increase in trapping. A reduction in the share of damaged fruits was observed in all trials, but with a generally low positive correlation with the number of catches, statistically significant only for the cone and bottle traps. The outcomes are discussed in view of the various factors determining mass trapping efficacy against flies of the family Tephritidae and the potential adoption of this control measure by farmers within an integrated pest management strategy, suitable to reduce the damage of the *R. batava* fly in organic sea buckthorn orchards, with an approach pursuing circular economy principles.

## Simple Summary

The growing interest of consumers in products obtained from sea buckthorn (*Hippophae rhamnoides* L.) has contributed to an increase in the area under cultivation in Poland and world-wide. The greatest threat to this crop is the sea buckthorn fruit fly (*Rhagoletis batava* Hering), which can destroy even the entire harvest. Due to the specific biology of the pest and the lack of registered chemical control agents, control is difficult. Monitoring and mass trapping are two measures of integrated pest management (IPM) that can contribute to reducing the fruit damage from this pest. After carrying out trials in several locations for up to five years, it was shown that mass trapping with a commercial trap containing an attractant developed for a similar fruit fly affecting other crops (*Ceratitis capitata*) and a homemade trap, prepared by recycling plastic bottles, containing a 4% solution of ammonium phosphate (a common fertilizer) effectively reduced *Rhagoletis batava* populations and their damage to fruits.

## 1. Introduction

Sea buckthorn (*Hippophae rhamnoides* L.) is a berry species that has long been used in traditional medicine in Europe and Asia [1] for the treatment of disturbances of the digestive and cardiovascular systems [2,3], among others, exploiting fruits, seed oils, and leaf extracts [4,5,6]. Currently, sea buckthorn has become an increasingly important raw material in the food industry [7] for producing juice or jams from the pulp and oil from seeds, used as an additive to enrich the composition of food products [8,9].

The growing demand for high-quality sea buckthorn fruits, specially produced in organic farming, has created an increased interest in the cultivation of this species in Poland in recent years, leading to an estimated land area of sea buckthorn orchards of about 120 ha. However, this has also led to a growing risk of pests’ occurrence, particularly the sea buckthorn fruit fly (*Rhagoletis batava* Hering, 1958), both in Poland and other European countries [10,11,12].

Despite the large yearly fluctuations in adult abundance, due to the species’ high susceptibility to biotic and abiotic factors, the high fertility of females (200 or more eggs/female) and their ability to undergo long-term diapause in unfavorable conditions make *R. batava* the most damaging pest of this crop, since it attacks only the fruit and can damage up to 100% of the crop [13]. *R. batava* larvae can overwinter in the soil for up to three years [14], and pupation occurs about 1 week before adults’ emergence, which depends on temperature [15]. In different regions where *R. batava* occurs, the sum of effective temperatures for the adults’ flight may vary, but it has been shown that 377.7 ± 15.5 °C degree days are a general value [16], counted from the day when the temperature reaches 10 °C. Egg laying only occurs on warm days, within 6–14 days of the start of flight, under the skin of the fruit. During the first 3–4 weeks of their development, the larvae feed on the fruit pulp, and, after destroying one fruit, they move on to the next in the cluster. Fruits colonized by larvae soften, gradually deform, and then dry up without falling off [15]. It should be noted that, even with minor damage to the crop, these berries contaminate the rest of the crop [16].

The control of *R. batava*, like other species of the Tephritidae family, can be based on chemical, physical, and biological methods [10,17,18]. However, a limitation in the methods derives from the biological cycle, with larvae spending their entire life cycle in the fruit pulp, which makes them less susceptible to chemical control measures, thus having to target mainly the imago stage, i.e., the fly. Moreover, the availability of active substances is also limited: in Poland, there are no registered active substances for controlling *R. batava* in sea buckthorn crops, either in conventional or organic crops [18,19].

Flies of the Tephritidae family, which overwinter under their hosts, show no motivation to move long distances, except when there is a lack of fruit on the host plant. The maximum dispersal flight distances are difficult to estimate but can vary from 100 to 500 m up to several km [10] within 24 h. Flies of the Tephritidae family are best caught in yellow traps [20], but *R. batava* flies react differently to the colors of traps used for monitoring and trapping, with the highest number of catches recorded on yellow traps [21]. In order to improve the attraction of fruit flies, lures can be used [22,23], and this strategy was suitable also in the case of *R. batava* flies, with attractants based on ammonium carbonate and acetate [24] which could be exploited also for mass trapping.

The present study aimed at identifying a suitable trap and attractant combination that could optimize the mass trapping of *R. batava* and support its control within a strategy of integrated management feasible particularly for organic orchards of sea buckthorn.

## 2. Materials and Methods

### 2.1. Location of Trials

The evaluation of traps and attractants for the mass trapping of *R. batava* was conducted on three sea buckthorn plantations: two in the Pomeranian Province (53°51′37″ N, 19°29′35″ E), where the plantations (Przezmark I and Przezmark II) were located approximately 3 km apart in a straight line, and one in the Lublin Province (Pereszczówka—51°55′32″ N, 22°55′58″ E). All orchards were cultivated with a spacing between trees of 2 m × 3.5 m (approx. 1400 trees per ha). In Pereszczówka, varieties of Russian origin were grown, while, in Przezmark, varieties of German origin were cultivated. An initial screening trial of the various traps and attractants was carried out in 2018 in Przezmark I. Following this, in 2019–2022, the trials were carried out in all three locations. A randomized block design with three replicates, each formed by a plot of about 0.2 ha, was applied for all trials. Additionally, a 3.5 m wide buffer zone was designated on each side of each plot where no treatments were carried out, and the fruits were always sampled from the central parts of the plot.

### 2.2. Characteristics of Traps and Attractants

#### 2.2.1. Traps

**Figure 1:**
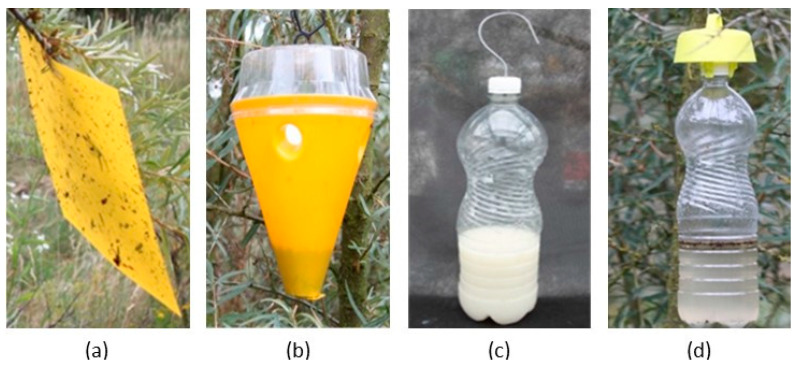
The four kinds of traps used for the trials: (**a**) sticky trap (T), (**b**) cone trap (C), (**c**) bottle (B), and (**d**) bottle with yellow cap (Bn).

Four types of traps were used in the experiments (Figure 1): a sticky chromotropic trap (T), considered the standard method for catching fruit flies, a cone trap (C), a bottle trap (B), and a bottle trap with a yellow cap (Bn). The sticky trap was a commercial product measuring 10 cm × 20 cm (Medchem, Stara Iwiczna, Poland). The cone trap was a commercial trap (Probodelt, Amposta, Spain) which consists of a plastic container with a cover coated with an insecticide (pyrethroid). The bottle traps were homemade traps using 1.5 L volume PET plastic bottles, in which four small holes (about 5 mm diameter) were drilled in their upper parts. The size of the holes was defined to allow free access for *R. batava* flies, while avoiding the entrance of large flies. The yellow cap of the Bn version was also a commercial product (Carello Roberto di Bellini Vanda, Pianezza, Italy). The control plots did not have any traps.

#### 2.2.2. Attractants

The following attractants were used in the experiments:

(1) A commercial attractant dedicated to the capture of *Ceratitis capitata* flies (C.C.)—(Probodelt, Amposta, Spain);
(2) A 4% aqueous solution of ammonium phosphate fertilizer with 18:20 N:P content (N) of about 0.5 L/bottle;
(3) A 4% aqueous solution of ammonia with the addition of European anchovy (*Engraulis encrasicolus*) in the amount of 1 piece/1 trap (AS);
(4) An aqueous solution of 2.2 g/L of sucrose to which 10 g of bread yeast (RD) was added;
(5) A proprietary hydroalcoholic solution in a specially prepared ampoule composed of ammonium acetate and trimethylamine hydrochloride, with the addition of putrescine dihydrochloride and ammonium carbonate—concentration/type A2 (BCHM Miśkiewicz, Warsaw, Poland) (A2);
(6) A proprietary hydroalcoholic solution with the same compounds used for attractant 5, but with a different concentration (BCHM Miśkiewicz, Warsaw, Poland) (A3).

### 2.3. Evaluation of the Trap Efficacy

A trap density rate per area of 80 traps/ha was used in each plantation. Traps were deployed in three rows: in two of them the traps were placed every 16.5 m and in one row every 20.0 m. The traps were hung on the plantations before the emergence of the fruit fly. During the season, the number of adults caught in all traps was counted with a 10-day frequency. At harvest, the degree of fruit damage was assessed on a random sample of 1200 fruits (two samples of 200 fruits collected from various trees of each replication).

### 2.4. Statistical Analysis

The data were analyzed using ANOVA, and means of separation were obtained by applying the Newman–Keuls test at a significance level of *p* = 0.05. Numerical data expressed as a percentage were first converted using Bliss’s transformation formula. Efficacy was calculated according to Abbott’s formula [25]. To determine the correlation between the number of flies caught and the efficacy expressed as a percentage of damaged fruit in relation to the control, Spearman’s rank-order correlation was performed at a significance level of *p* ≤ 0.05. In addition, the coefficient of variation (CV) was calculated to define the overall variability of the values related to the damaged fruits in a given orchard and year (i.e., without allocating them to the specific treatment), which was used as a proxy to evaluate the overall changes in the *R. batava* population (total pest population—TPP) present in the orchard. Large values of CV represent high relative dispersions of the data around the mean [26]. CV was calculated using the following formula:

$$CV=\sigma /\bar{x}$$

where $\bar{x}$ is the arithmetic mean of the population and *σ* is the standard deviation of the population. The population variability was considered low when *CV* ranged between 0 and 20, average when *CV* = 20–40, high when *CV* = 40–60, and very high when *CV* > 60.

Statistical calculations were performed using Statistica ver. 13.1.

## 3. Results

### 3.1. Preliminary Screening of Traps and Attractants

**Figure 2:**
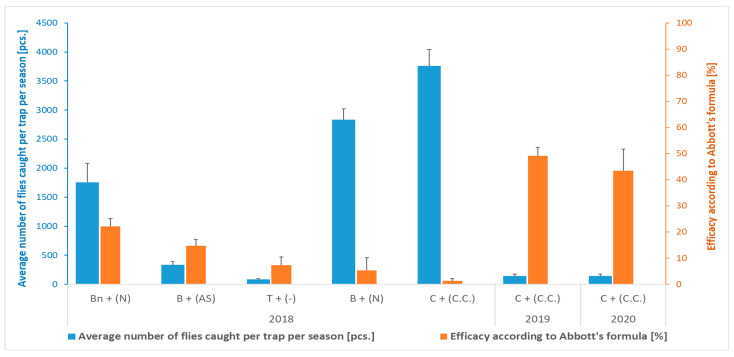
Average number of *Rhagoletis batava* flies caught per trap per season and efficacy in reducing fruit damage according to Abbott’s formula (Przezmark I). Mean ± SEM. T—chromotropic sticky trap; Bn or B—bottle trap with or without yellow cap, respectively, with an attractant based on ammonium phosphate solution (N) or ammonia solution and anchovy (AS); C—cone trap with an attractant for *Ceratitis capitata* (C.C.).

**Figure 3:**
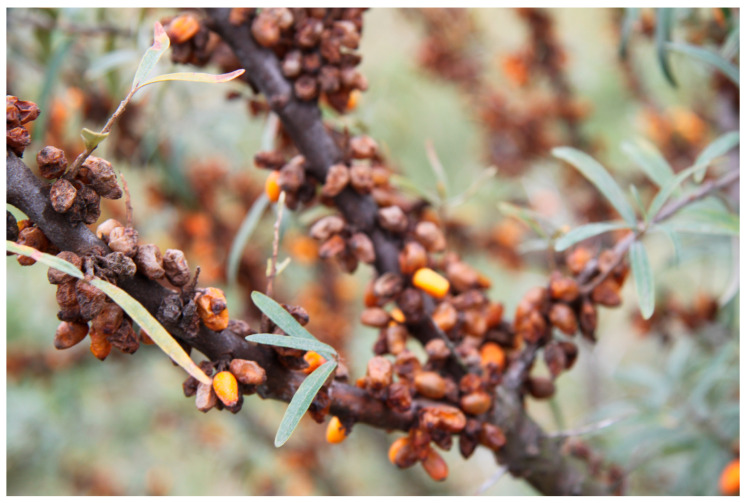
The effect of the damage caused by *Rhagoletis batava* on the fruits of sea buckthorn in the Przezmark trial. Damaged fruits appear brownish or black, while undamaged fruits are those with yellow color.

The number of flies caught by the various types of traps in combination with the relevant attractants during the 2018 season in the location Przezmark I varied between 82.7 (sticky traps) and 3758.1 (cone trap with attractant for *C. capitata*) (Figure 2). However, the efficacy in reducing fruit damage was generally very low, though statistically significant, in comparison to the control plots (without traps) (Table 1 and Figure 2), and not correlated with the number of catches. Indeed, in the control plots (without traps), practically all the fruits collected were damaged (Figure 3), a condition that the farmer had already observed in the season preceding the experiments, prompting his search for a solution. Moreover, a low CV of the value of damaged fruits (Table 1) indicated little variation within the orchard, irrespective of the presence of traps.

### 3.2. Mass Trapping

**Figure 4:**
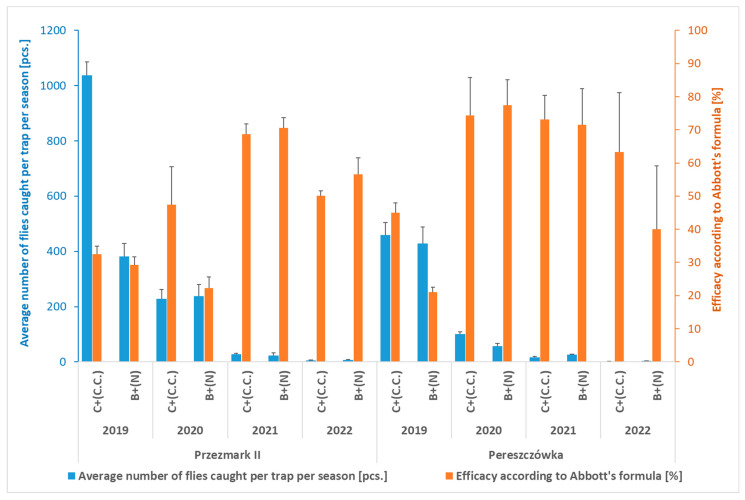
Average number of *Rhagoletis batava* flies caught per trap per season and efficacy in reducing fruit damage according to Abbott’s formula (Przezmark II and Pereszczówka, 2019–2022). Mean ± SEM. Bottle traps with an attractant based on ammonium phosphate solution (B + N) and cone traps with an attractant for *Ceratitis capitata* (C + C.C.).

Based on the trial carried out in 2018, traps with a relative high efficacy in attracting the flies were selected for further mass trapping studies, i.e., the cone trap with attractant C.C., used for two years on the same orchard (Przezmark I—Table 1 (2019–2020) and Figure 2), and the bottle traps with the ammonium phosphate solution (N), which were used for four seasons (2019–2022) in two other orchards (Pereszczówka and Przezmark II (Table 2 and Figure 4)).

In the following years, at the Przezmark I location, the deployment of the cone traps resulted in almost halving the percentage of damaged fruits compared to the control (Table 1). In both years, a similarly low number of flies were caught in the traps (Figure 2). The CV of the damaged fruit data in this trials ranged from 20 to 40% in both years, which indicated an average variability of the data.

The same level of efficacy of the cone traps was also observed during the four-year-long trial carried out in the other two locations (Table 2 and Figure 4). Interestingly, the bottle traps with the ammonium phosphate attractant caught a similar average number of flies in both trials (Figure 4). This resulted, for both kinds of traps, in a significant reduction in the percentage of damaged fruit compared to the control (Table 2). In these trials, the CV of the data related to damaged fruits, similarly to the Przezmark I site, increased along the seasons (Table 2).

**Figure 5:**
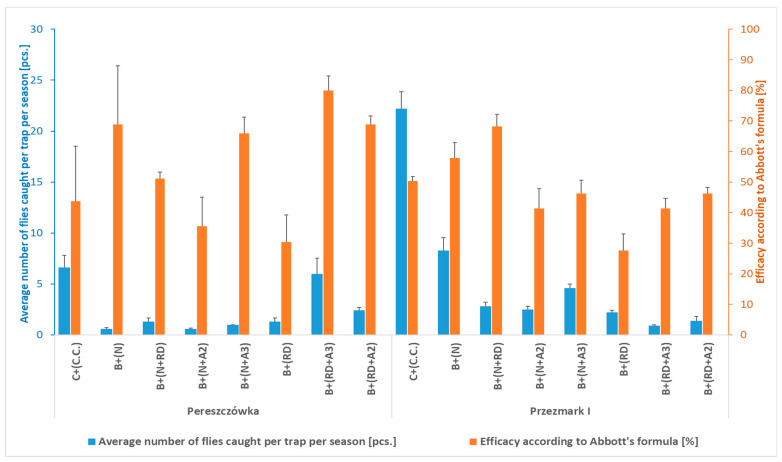
Average number of *Rhagoletis batava* flies caught per trap and season, and efficacy in reducing fruit damage according to Abbott’s formula (Pereszczówka and Przezmark I, 2022). Mean ± SEM. B—bottle trap with an attractant based on ammonium phosphate solution (N) and/or with sucrose and yeast solution (RD) and/or a proprietary hydroalcoholic solution of various compounds at different concentrations (A2, A3); C—cone trap with an attractant for *Ceratitis capitata* (C.C.).

To further increase the efficacy of bottle traps, considering their inexpensiveness and ease of adoption by farmers, in 2022, new attractants (RD, A2, and A3) were used in combination with the ammonium phosphate solution (N) and compared to the cone + (C.C.) and bottle + (N) traps, which had been tested in the previous years. In both orchards, a relatively small number of *R. batava* flies were caught during the season (0.6–22.2 per trap) (Figure 5). However, the efficacy in reducing the fruit damage of all kinds of trap and attractant combinations was significantly higher compared to the control. The lowest efficacy was determined for the B + (RD) trap (on average 29.0% for the two locations). Interestingly, the same trap/attractant enriched with the two new proprietary mixtures of attractants (A2 and A3) or with the ammonium phosphate solution (N) showed a higher efficacy (from 50 to 100%) than the trap containing only the yeast in both orchards. However, the addition of the A2 and A3 attractants to bottle traps containing the ammonium phosphate solution (N) did not increase their efficacy compared to those with only the solution (Table 3 and Figure 5). The CV related to the damaged fruit data of the two locations differed, being low in Pereszczówka and average in Przezmark I (Table 3).

### 3.3. Assessing the Relation Between Trapped Flies and Damaged Fruits

**Figure 6:**
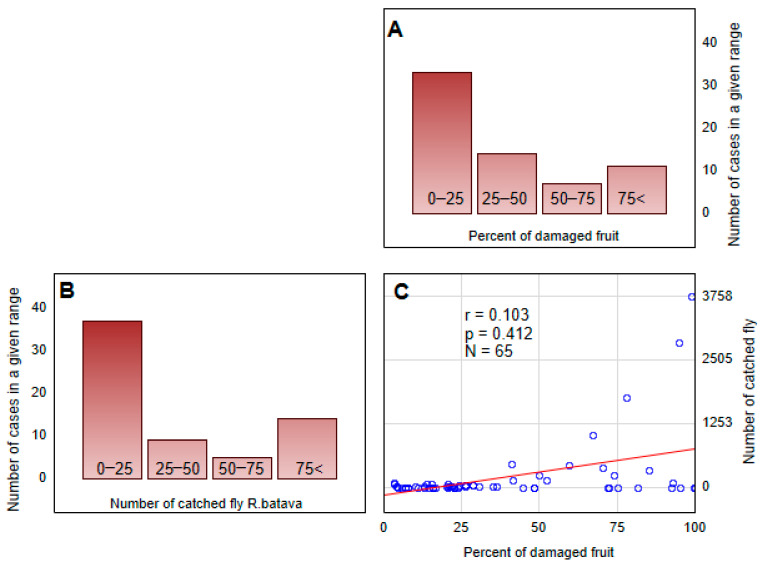
The frequency of (**A**) the percentage of damaged fruits and (**B**) the number of *Rhagoletis batava* flies caught by all kinds of traps. The correlation between these two datasets is also presented (**C**).

The correlation analysis between the number of *R. batava* flies caught and the percentage of damaged fruits showed a generally low positive correlation (Figure 6). However, for some specific trap/attractant types, i.e., the cone trap + (C.C.) (R2 = 0.76) and bottle trap + (N) (R2 = 0.66), the relation was statistically significant (Table 4).

## 4. Discussion

The development of organic methods for controlling the population of *R. batava* in sea buckthorn crops is particularly important in Poland due to the increasing economic interest in the crop. The solutions used so far have not been sufficiently effective, resulting in crop losses of up to 100% and very often leading to the elimination of the orchard due to a lack of profitability. Recommended agrotechnical methods [27], such as the use of cultivation treatments in tree rows and the sowing of perennial grasses on plantations as a factor limiting the *R. batava* population, did not bring the expected results due to the high damage caused by even a small number of these pests [10,15]. The use of soil cover in orchards to hinder the flight of Tephritidae flies is an effective method of reducing their numbers [18], but it is not fully economically justified for large orchards due to the high material and labor costs. Other biotic factors that have been found to contribute to the regulation of various tephritid populations, e.g., parasitoid wasps [28] or predatory ants [29] and entomopathogens [30], are not known to be so active in the case of *R. batava*. This could have also contributed to its steady territorial movement from Siberia toward Europe [13]. Therefore, in the absence of registered active substances, the mass trapping of *R. batava* flies seems to be the only possible control measure.

The low efficacy of the various traps tested in this study, which was observed during the first trial season in Przezmark I, could be justified by the very high incidence of *R. batava* in the orchard, where damage occurred already in previous years. It was indeed such high damage that prompted the farmer to look for advice for its control: the level of damage was confirmed during the preliminary trial by the complete destruction of the crop on the plots not having traps (control treatment with all fruits damaged). The high incidence of damaged fruit also in plots where a large number of flies were caught could be due to the high fertility of the R. batava female, being able to lay about 200 eggs, and the capacity of hatched larvae, unlike other fly species, to move from fruit to fruit [13]. However, it could also be hypothesized that the presence of traps and attractants may have caused an increased influx of flies from other parts of the orchard or nearby orchards, which would be consistent with observations about flies’ movement reported in other studies [10,15].

Mass trapping was then implemented for up to 5 years in the Przezmark I orchard and for a lower time frame in the other locations, using various traps and attractants. A reduction in the flies’ population, according to the number of adults trapped, was evident, even when taking into account possible fluctuations due to environmental and climatic factors, as well as host fruit abundance and availability [31,32,33]. The observed reduction in both the number of damaged fruits and the number of *R. batava* flies caught by traps, statistically proven for two trap/attractant combinations, is thus hypothesized to be the result of a stabilization of the overall pest population due to the continuous use of mass traps during the subsequent growing seasons. A similar trend was observed with the rose fruit fly (*Rhagoletis alternata*) on Damascus rose plantations and with *R. cerasi* [34,35]. However, despite such a significant reduction, which amounted to approximately 90% after applying the measure for 5 years, the level of fruit damage remained still high, above 20%. This confirmed the high harmfulness and difficulty of limiting the damage caused by *R. batava* [15], as well as by other fruit fly species [36,37,38,39], even in the presence of small pest populations, prompting the need of adopting an integrated, area-wide, and ecologically based scheme for the effective control of the pest [40], an approach which can benefit also from the introduction of mass trapping [41].

Several factors are key in determining mass trapping efficacy against flies of the family Tephritidae: attractant efficacy and durability, the size of the holes in the traps, the number and type of traps used and their placement in the field, the pest pressure, the degree of crop isolation, and the climatic conditions [42]. In case of *R. batava*, traps should be placed in the sunniest parts of the tree canopy [14], so the deployment of the trap on the tree may also affect its efficacy. The factors that could have been controlled, particularly the last one, were considered in the field trials of this study to assure the achievement of the highest possible efficacy.

The attractants used in the trials showed a varying efficacy to attract *R. batava* flies. The attractants based on ammonia-releasing chemicals (e.g., the ammonium phosphate solution) were effective in fly capture, resulting also in the highest efficacy in reducing fruit damage. Numerous studies have confirmed that ammonia-based attractants can improve the efficacy in catching flies of the family Tephritidae. Özdem and Kılınçer [34] achieved the best results in catching *R. cerasi* using yellow traps in combination with an ammonia lure. Similarly, the best result in capturing *Rhagoletis indifferens* and *R. cerasi* was obtained using yellow sticky traps and cylindrical traps in combination with ammonium carbonate [43], or using attractants based on ammonium acetate for *R. cerasi* [35,44]. However, the economic potential of attractants containing yeast, ammonium acetate, and trimethylamine in the mass trapping of *R. cerasi* flies was not confirmed [10], while, in the present study, the proprietary mixtures containing also these kinds of compounds were suitable for the mass trapping of *R. batava*.

The trials pointed out the possibility of using attractants developed for the capture of *C. capitata* also for the mass capture of other fruit fly species, including *R. batava*, though with a lower efficacy in reducing crop damage in sea buckthorn orchards compared to that recorded in citrus fruit crops [42]. This difference could be related to the high aggressiveness and harmful capacity of even small populations of *R. batava* [14,15].

The adequate density of traps deployed to the crop is another factor affecting the overall efficacy of reducing the pest damage. In the present study, the equivalent of 80 traps per hectare (i.e., 16 per replicate plot) were deployed, which was considered technically feasible considering the number of plants per hectare (about 1400) and, at the same time, likely to provide a sufficient density for effective trapping (1 trap for every 17 plants). The number of catches, particularly those of the initial trial, would confirm the correctness of the approach, considering that the trap density for the mass trapping of Tephritidae flies varies greatly. For example, the recommended density for the mass trapping of *C. capitata* ranged from 30 to 50 [41] to about 100 [45] or 165 [46], or 120 in the case of bottle traps [47]. For the mass trapping of *R. cerasi*, Daniel and Grudner [17] used between 200 and 800 traps/ha, depending on the type and kind of attractant. However, in the case of sea buckthorn, the cultivation system, which involves cutting all fruit-bearing shoots from the tree at harvest with weak fruiting in the following year, inducing the flies to move, i.e., provoking an uneven distribution of the *R. batava* population in the orchard, may be an additional factor to account for the observed limited correlation between the trapping efficacy and the fruit damage reduction. Nevertheless, it should be noted that, in all trials, the coefficient of variation in the data related to adult catches was lower at the beginning of the trials compared to following years, indicating a differentiation in the population across the treatments and thus substantiating the efficacy of the traps.

Considering the decline in the total number of flies caught after a five-year-long deployment period, the suggestion of Broumas et al. [48] to reduce the number of traps deployed as the pest population declines could make this crop protection measure appealing to farmers. Indeed, the economic factor is generally key to supporting the adoption of any agronomical measure. However, in the case of *R. batava*, which is difficult to completely control, particularly under organic farming, the economic factor becomes frequently prevalent for the farmer’s decision. The results, confirming previous works [49], obtained with the home-made, recycled plastic bottle traps filled with attractants based on an aqueous solution of a common fertilizer (ammonium phosphate) and/or an aqueous solution of sugar with the addition of yeast make the use of this type of trap financially encouraging, and also in line with the real application of the principles of a circular economy at the farm level [50].

## 5. Conclusions

Trials carried out under diverse environmental and agronomical conditions, testing various traps and attractants for the mass trapping of *R. batava* adults in organic sea buckthorn orchards, demonstrated the possibility of reducing the population size of the pest, significantly limiting the damage to the crop fruits. Inexpensive traps that can be directly prepared by the farmer using recycled plastic bottles filled with an easy-to-prepare solution of a common fertilizer assured a comparable efficacy to commercial traps developed for *C. capitata*. The overall efficacy after up to five years of trap deployment was still not sufficient to fully protect the crop from this very aggressive and harmful pest. The adoption of an integrated pest management strategy, possibly over wide areas, with the simultaneous deployment of mass trapping and application of other agrotechnical treatments (e.g., cover crops with perennial grasses without mowing them during the period when flies emerge from the soil, or soil treatment with entomopathogenic fungi targeting the juvenile forms of the pest) could increase the efficacy in reducing the fruit damage, particularly in organic sea buckthorn orchards.

## Figures and Tables

**Table 1:** The average number of fruits damaged by *Rhagoletis batava* flies after a preliminary trial testing various traps (2018) and with the deployment of the cone trap in the following seasons in the orchard located in Przezmark (Przezmark I). Mean ± SD.

Type of Trap + (Attractant) ^$^	Average Number of Damaged Fruits * [%]		
	2018	2019	2020
T (none)	92.7 ± 7.9 b	-	-
Bn + (N)	77.8 ± 11.8 a	-	-
B + (AS)	85.2 ± 5.7 a	-	-
B + (N)	94.7 ± 7.4 b	-	-
C + (C.C.)	98.7 ± 2.0 bc	41.5 ± 6.7 a	52.2 ± 17.3 a
Control (without traps)	100.0 ± 0.0 c	81.5 ± 3.1 b	92.3 ± 5.6 b
F(df_1_, df_2_)	F(5, 30) = 11.377	F(1, 10) = 177.117	F(1, 10) = 33.321
*p*-value	*p* < 0.001	*p* < 0.001	*p* < 0.001
ŋ^2^	ŋ^2^ = 0.655	ŋ^2^ = 0.946	ŋ^2^ = 0.769
Mean ± SD	91.5 ± 10.0	61.5 ± 20.6	72.2 ± 23.2
*CV*	11.0	33.4	32.2

^$^ T—chromotropic sticky trap; Bn or B—bottle trap with or without yellow cap, respectively, with an attractant based on ammonium phosphate solution (N) or ammonia solution and anchovy (AS); C—cone trap with an attractant for *Ceratitis capitata* (C.C.). * Values marked with different letters differ from each other at *p* ≤ 0.05 significance level.

**Table 2:** Average number of fruits damaged by *Rhagoletis batava* flies as affected by the deployment of the cone and bottle traps in two locations in the seasons for 2019–2022.

Type of Trap + (Attractant) ^$^	Average Number of Damaged Fruits * [%]			
	2019	2020	2021	2022
	Przezmark II			
C + (C.C.)	67.2 ± 5.7 a	49.8 ± 26.4 a	21.8 ± 2.6 a	20.3 ± 2.8 a
B + (N)	70.3 ± 5.8 a	73.8 ± 7.3 b	20.8 ± 4.9 a	24.0 ± 2.5 a
Control (without traps)	99.5 ± 0.8 b	95.0 ± 1.9 c	72.0 ± 12.4 b	48.3 ± 6.6 b
F(df_1_, df_2_)	F(2, 15) = 148.467	F(2, 15) = 15.396	F(2, 15) = 62.607	F(2, 15) = 72.997
*p*-value	*p* < 0.001	*p* < 0.001	*p* < 0.001	*p* < 0.001
ŋ^2^	ŋ^2^ = 0.952	ŋ^2^ = 0.672	ŋ^2^ = 0.893	ŋ^2^ = 0.907
Mean ± SD	79.0 ± 15.2	72.9 ± 23.5	38.3 ± 25.0	30.9 ± 13.0
CV	19.2	32.2	65.4	42.3
	Pereszczówka			
C + (C.C.)	41.0 ± 2.3 a	3.3 ± 3.3 a	4.0 ± 1.9 a	7.0 ± 7.2 a
B + (N)	59.3 ± 5.6 b	3.2 ± 2.0 a	4.0 ± 3.0 a	12.7 ± 7.5 ab
Control (without traps)	75.2 ± 6.4 c	15.8 ± 3.6 b	16.7 ± 3.7 b	22.5 ± 3.7 b
F(df_1_, df_2_)	F(2, 15) = 61.783	F(2, 15) = 21.497	F(2, 15) = 20.214	F(2, 15) = 6.580
*p*-value	*p* < 0.001	*p* < 0.001	*p* < 0.001	*p* < 0.01
ŋ^2^	ŋ^2^ = 0.892	ŋ^2^ = 0.741	ŋ^2^ = 0.729	ŋ^2^ = 0.467
Mean ± SD	58.5 ± 14.7	7.4 ± 6.5	8.2 ± 6.6	14.0 ± 8.6
CV	25.1	88.0	79.8	61.5

^$^ Bottle traps with an attractant based on ammonium phosphate solution (B + N) and cone traps with an attractant for *Ceratitis capitata* (C + C.C.). * Values marked with different letters differ from each other at *p* ≤ 0.05 significance level.

**Table 3:** Average number of fruits damaged by *Rhagoletis batava* flies as affected by the deployment of different kinds of traps in two locations in 2022. Mean ± SD.

Type of Trap ^$^ + (Attractant)	Average Number of Damaged Fruits * [%]	
	Pereszczówka	Przezmark I
B + (N)	7.0 ± 7.4 a	20.3 ± 2.5 ab
C + (C.C.)	12.7 ± 7.2 ab	24.0 ± 2.8 b
B + (N + RD)	11.0 ± 1.7 ab	15.3 ± 3.8 a
B + (N + A2)	14.5 ± 3.0 bc	28.3 ± 5.8 b
B + (N + A3)	7.7 ± 3.3 a	26.0 ± 7.5 b
B + (RD)	15.7 ± 2.5 bc	35.0 ± 5.8 c
B + (RD + A3)	4.5 ± 1.9 a	28.3 ± 6.4 b
B + (RD + A2)	7.0 ± 0.6 a	26.0 ± 3.5 b
Control (without traps)	22.5 ± 3.7 c	48.3 ± 6.6 d
F(df_1_, df_2_)	F(8, 45) = 7.5692	F(8, 45) = 18.271
*p*-value	*p* < 0.001	*p* < 0.001
ŋ^2^	ŋ^2^ = 0.574	ŋ^2^ = 0.765
Mean ± SD	11.4 ± 6.5	27.2 ± 10.2
CV	57.2	47.7

^$^ B—bottle trap with an attractant based on ammonium phosphate solution (N) and/or with sucrose and yeast solution (RD) and/or a proprietary hydroalcoholic solution of various compounds at different concentrations (A2, A3); C—cone trap with an attractant for *Ceratitis capitata* (C.C.). * Values marked with different letters differ from each other at *p* ≤ 0.05 significance level.

**Table 4:** Table of correlation statistics between the number of *Rhagoletis batava* flies caught and the percentage of damaged fruit for each type of trap.

Type of Trap + (Attractant) ^$^	Sample Size (N) **	Spearman’s R Coefficient	Value of the t-Statistic t (*n* − 2)	Probability Value (*p*)
C + (C.C.)	16	0.76 *	4.32	0.0007
B + (N)	24	0.66 *	4.06	0.0005
B + (RD)	6	0.54	1.29	0.2657
B + (N + RD)	Too small sample size			
T				

^$^ B—bottle trap with an attractant based on ammonium phosphate solution (N) and/or with sucrose and yeast solution (RD); C—cone trap with an attractant for *Ceratitis capitata* (C.C.); T—chromotropic sticky trap. * Values significant at *p* ≤ 0.05 significance level. ** Number of samples for a given trap type from all experiments.

## Data Availability

The original contributions presented in this study are included in the article. Further inquiries can be directed to the corresponding authors.

## Abbreviations

The following abbreviations are used in this manuscript:

SD	Standard Deviation
SEM	Standard Error of the Mean
